# Left ventricular perforation following impella® CP placement in a resuscitated STEMI patient with cardiogenic shock: a rare complication and case report

**DOI:** 10.1093/ehjcr/ytag050

**Published:** 2026-01-28

**Authors:** Omar Hajji, Mohammad Abumayyaleh, Tobias Schupp, Michael Behnes, Ibrahim Akin

**Affiliations:** Department of Cardiology, Haemostaseology and Medical Intensive Care, University Medical Center Mannheim, Medical Faculty Mannheim, Heidelberg University, Theodor-Kutzer-Ufer 1-3, 68167 Mannheim, Germany; Department of Cardiology, Haemostaseology and Medical Intensive Care, University Medical Center Mannheim, Medical Faculty Mannheim, Heidelberg University, Theodor-Kutzer-Ufer 1-3, 68167 Mannheim, Germany; Department of Cardiology, Haemostaseology and Medical Intensive Care, University Medical Center Mannheim, Medical Faculty Mannheim, Heidelberg University, Theodor-Kutzer-Ufer 1-3, 68167 Mannheim, Germany; Department of Cardiology, Haemostaseology and Medical Intensive Care, University Medical Center Mannheim, Medical Faculty Mannheim, Heidelberg University, Theodor-Kutzer-Ufer 1-3, 68167 Mannheim, Germany; Department of Cardiology, Haemostaseology and Medical Intensive Care, University Medical Center Mannheim, Medical Faculty Mannheim, Heidelberg University, Theodor-Kutzer-Ufer 1-3, 68167 Mannheim, Germany

**Keywords:** Case report, Impella, STEMI, Cardiogenic shock, Left ventricular perforation, Device-related complications, Mechanical circulatory support

## Abstract

**Background:**

The use of mechanical circulatory support (MCS) for acute haemodynamic stabilization in cardiogenic shock has increased over the past decade. Impella® heart pumps (Abiomed) are intravascular microaxial blood pumps designed to provide temporary MCS during high-risk percutaneous coronary intervention (HRPCI) and the management of cardiogenic shock. However, despite their increasing use, there are limited randomized clinical trials to support the benefits of the therapy and growing concern regarding complication rates. The objective of this report is to present a rare case of a left ventricular perforation after Impella® CP placement in a resuscitated patient after ST-elevation myocardial infarction (STEMI).

**Case summary:**

We present the case of a 54-year-old patient who suffered an out-of-hospital cardiac arrest (OHCA) due to STEMI and was successfully resuscitated. Due to persistent cardiogenic shock, Impella® CP support was initiated. The clinical course was complicated by recurrent and refractory ventricular fibrillation (VF), requiring multiple resuscitations in the intensive care unit (ICU). These resuscitative efforts, combined with friable necrotic myocardial tissue from the infarction, contributed to a left ventricular perforation and dislocation of the Impella device in the pericardium. To stabilize the patient, veno-arterial extracorporeal membrane oxygenation (VA-ECMO) was initiated, followed by transfer to the cardiac surgery department. Despite the severity of the complications, the Impella was successfully explanted and replaced with a transaxillary Impella® 5.5 pump without the need for open-heart surgery. Extubation was achieved few days later, and the patient demonstrated progressive clinical recovery with successful weaning from the ECMO as well as the Impella 5.5.

**Conclusion:**

This case demonstrates that profound myocardial fragility following resuscitated STEMI may be a critical co-factor for Impella-related perforation, underscoring the necessity for extreme procedural vigilance and timely, multidisciplinary management in patients requiring mechanical circulatory support.

Learning points

- Percutaneous microaxial flow pump devices are increasingly used for mechanical circulatory support to enhance haemodynamics.
- Reported complications include vascular issues (bleeding, acute limb ischaemia) and other risks such as cardiac perforation, mitral chordae rupture, and stroke.
- Robust data from randomized trials are needed to better define the risks and benefits and guide appropriate clinical use.

## Introduction

Cardiogenic shock (CS) is a life-threatening condition characterized by inadequate tissue perfusion due to severe cardiac dysfunction that occurs in approximately 8% to 10% of patients with ST-segment elevation myocardial infarction (STEMI).^1,2^ Despite advancements in medical and interventional therapy, mortality rates remain high, approximating 40% to 50%.^1,3^ Over the past decade, the use of mechanical circulatory support (MCS) devices in CS and high-risk percutaneous coronary intervention (HRPCI) has grown.^4^ While the use of intra-aortic balloon pump (IABP) has significantly decreased, the use of intravascular microaxial left ventricular assist devices (LVADs) increased substantially among patients suffering acute myocardial infarction (AMI) complicated by CS.^5^ The Impella® CP (Abiomed, Danvers, MA, USA) is a percutaneously inserted, catheter-based microaxial pump designed to provide temporary left ventricular (LV) unloading and support systemic circulation in patients with severe LV failure.^6^ The Impella® CP is inserted via the femoral artery and advanced retrogradely across the aortic valve into the LV, where it directly pumps blood from the LV into the ascending aorta at a maximum flow rate of approximately 3.5–4.3 L/min.^4^ By reducing LV end-diastolic pressure and myocardial oxygen demand while improving cardiac output and coronary perfusion, the Impella® CP plays a crucial role in managing CS secondary to AMI.^7,8^ However, its placement is associated with procedural and device-related complications, including vascular injuries, haemolysis, thromboembolic events, arrhythmias, and, in rare cases, structural damage to the heart, such as left ventricular perforation.^9,10^ We report the clinical presentation and management of a patient who developed LV perforation following placement of an Impella® CP device in the setting of a CS due to acute coronary syndrome (ACS).

## Summary figure

**Table:** 

Timeline	Events
Day 0: prehospital phase	Chest pain and suspected STEMI: Patient transported directly to the catheterization laboratory. Initial ECG: ST-segment elevations in leads V2–V5. OHCA: Ventricular fibrillation occurred. Defibrillation: was performed, successfully converting VF to sinus rhythm. Medications: ASA and heparin administered. Patient was conscious at hospital arrival.
Day 0: hospital arrival and resuscitation	Recurrent VF: Patient deteriorated into persistent ventricular fibrillation. CPR initiated: Including intubation and mechanical ventilation. Emergency coronary angiography during CPR: showing complete occlusion of the LAD. PCI and Two DES implanted. ROSC: Achieved after 23 min of resuscitation.
Day 0: cardiogenic shock mManagement	Left ventricular dysfunction and Impella CP implantation for haemodynamic support. Persistent refractory VF, despite all therapeutic measures. Prolonged CPR. VA-ECMO initiated: last-resort intervention. CT scan: revealed left ventricular free wall perforation and minimal pericardial effusion.
Day 1: heart surgery transfer	Transfer to cardiac surgery department at another UMC. Impella removal without open-heart surgery. Inferior pericardiotomy performed: Drainage system placed. Surgical Impella 5.5 implanted: For continued LV support. Persistent ST-elevation and akinesia: concern for in-stent thrombosis. Second catheter intervention and recanalization of the thrombosed LAD stent.
Day 1–3: recovery and weaning from support	Progressive haemodynamic improvement & recovery.
Day 4	ECMO removal
Day 5	Extubation
Day 6	Impella removal
Day 7 and further	Transfer back to original institution. Stable ICU course with LVEF improvement (41% at discharge).
Follow-up at 8 weeks	Heart Failure guideline-directed medical therapy good tolerated No ventricular arrhythmias recorded by the WCD. LVEF of 43% on cardiac MRI with no thrombus or residual shunt. Coronary angiography confirmed good postinterventional result.

## Case presentation

A 54-year-old male patient, with no relevant medical history, was transported directly to our catheterization laboratory due to retrosternal chest pain and suspected ST-elevation myocardial infarction (STEMI). The chest pain had begun approximately 1 h before hospital arrival. The initial electrocardiogram (ECG) showed ST-segment elevations in leads V2–V5. Prehospital defibrillation was performed by the emergency medical team due to ventricular fibrillation (VF), resulting in immediate conversion to sinus rhythm with the patient regaining consciousness. Prehospital loading consisted of acetylsalicylic acid (ASA) 250 mg i.v. and unfractionated heparin 5000 IU i.v.

Upon arrival at the hospital, the patient developed recurrent VF, which progressed to a persistent state. Guideline-based resuscitation was initiated with intubation and mechanical ventilation, and emergency coronary angiography was performed under ongoing mechanical CPR (Corpuls® CPR). The procedure revealed an occlusion of the left anterior descending artery (LAD), necessitating percutaneous coronary intervention (PCI) with two drug-eluting stents (DES) (*cf. supplementary complement*). This intervention resulted in the return of spontaneous circulation (ROSC) after 23 min of resuscitation. Despite ROSC, the patient remained in CS with severely reduced left ventricular ejection fraction (LVEF). Consequently, an Impella® CP device was implanted in the catheterization laboratory for haemodynamic support.

The Placement was performed under continuous fluoroscopy with a contralateral pigtail catheter used as a landmark for the left-ventricular apex. The device was advanced over a 0.018-inch Hi-Torque wire; transoesophageal echocardiography was not available at implantation. Initial console parameters were *P*-level 7, purge pressure 530 mmHg, purge flow 4.7 mL·h⁻¹. No alarms were recorded during the first 30 min of support, and the device was neither repositioned nor replaced prior to the index perforation.

Admission biomarkers were markedly elevated: high-sensitivity troponin-I 5.493 μg/L (reference 0–0.045 μg/L), lactate dehydrogenase (LDH) 518 U/L (reference 120–246 U/L), and CK-MB 86 U/L (reference 0–24 U/L), consistent with a large acute anterior infarction.

**Figure 1:**
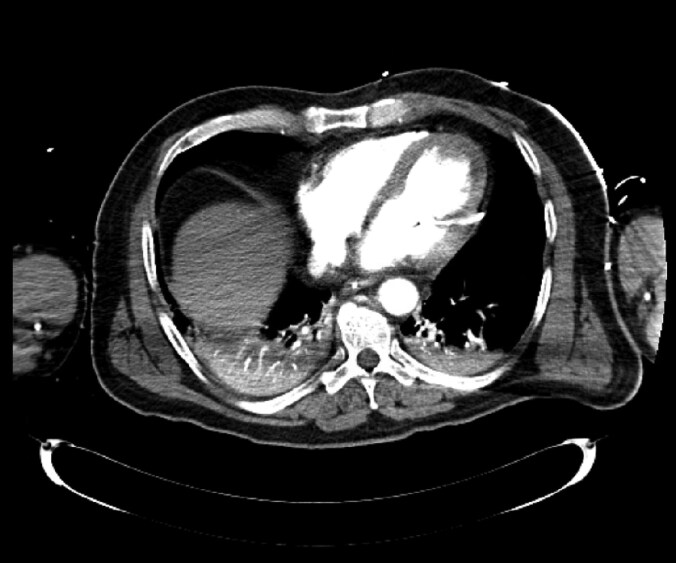
Axial CT scan demonstrating left ventricular (LV) perforation secondary to Impella® CP placement.

**Figure 2:**
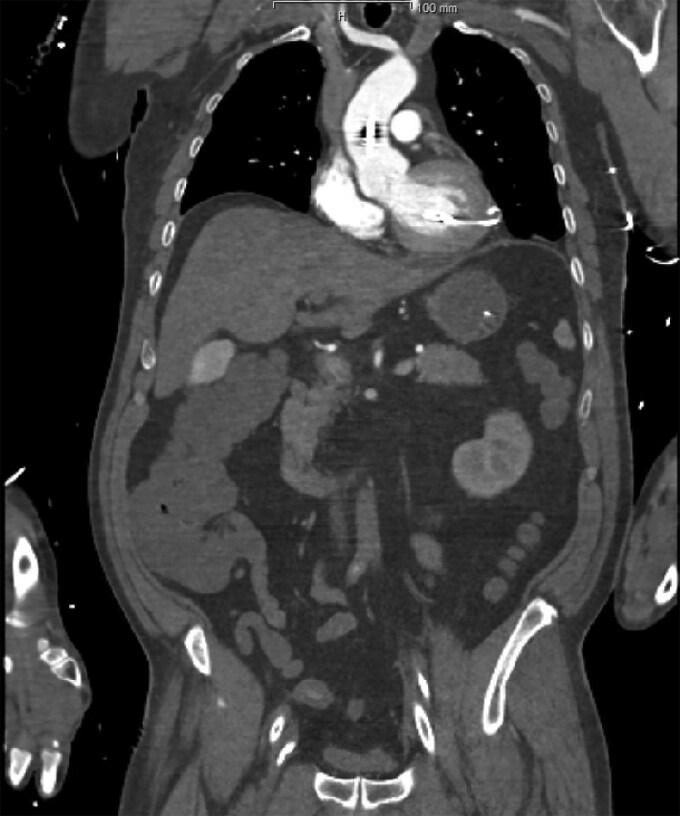
Coronal CT scan illustrating LV perforation caused by the Impella® CP device.

The patient was subsequently transferred to the cardiac intensive care unit (ICU). An initial bedside echocardiogram showed no pericardial effusion and confirmed that the device was correctly positioned in the left ventricle. However, refractory VF persisted despite all therapeutic measures, leading to prolonged CPR. Ultimately, veno-arterial extracorporeal membrane oxygenation (VA-ECMO) was initiated as a last-resort intervention. Following ECMO initiation and in accordance with our internal standard operating procedure (SOP), a computed tomography (CT) scan was performed, revealing a perforation of the left ventricle free wall, resulting in a minimal pericardial effusion *(Figure 1, 2).* Additionally, an abnormality in the position waveform was observed on the Impella® console display, showing a flattened, dampened curve with loss of the typical biphasic flow signal.

The patient was transferred to the cardiac surgery department at another university medical centre, where the Impella device was successfully removed without the need for open-heart surgery. Simultaneously, a drainage system was placed following an inferior pericardiotomy, and a surgical Impella® 5.5 was implanted for continued left ventricular support. Due to persistent ST-segment elevation on ECG, akinesia of the anterior left ventricular wall, and concern for in-stent thrombosis, a second catheter intervention was performed. This procedure successfully recanalized the thrombosed LAD stent.

Over the following days, the patient exhibited progressive haemodynamic improvement, allowing for the gradual weaning of ECMO and Impella®. ECMO was explanted on Day 4, and the Impella® was removed on Day 6. After extubation, the patient showed continuous recovery and was subsequently transferred back to our institution. The patient remained stable in the ICU under close monitoring, with an improvement in LVEF over the next few days. At discharge, three-dimensional transthoracic echocardiography *(TTE; Video 4 in the Supplement)* showed an LVEF of 41%.

### Follow-up

**Figure 3:**
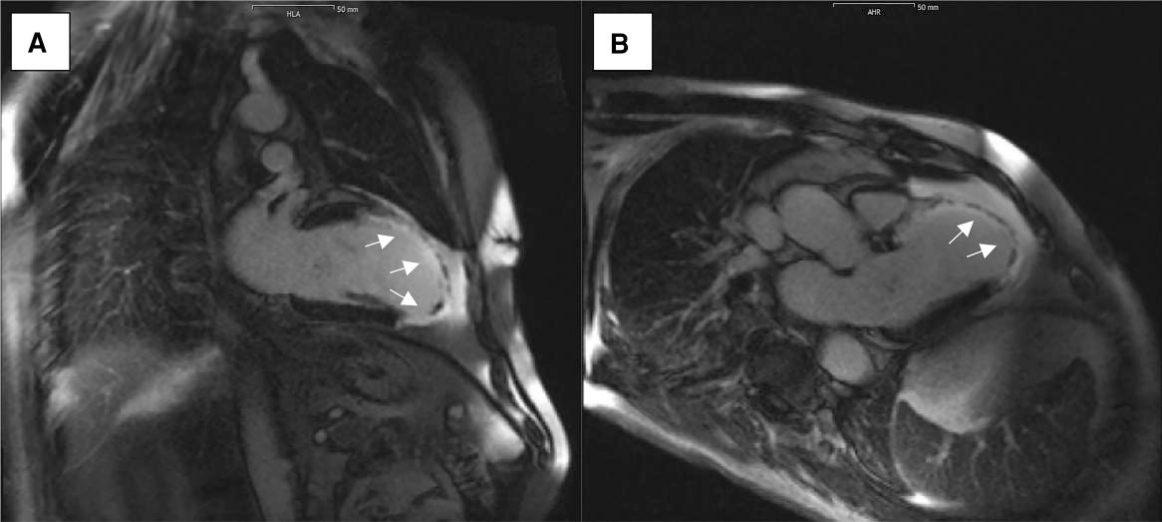
CMRI in two-chamber (*A*) and three-chamber (*B*) views demonstrating late gadolinium enhancement in the left anterior descending coronary artery territory.

After a 4-week cardiac-rehabilitation programme, the patient returned to our heart-failure clinic 60 days after the index STEMI. He reported 100% adherence to the prescribed wearable cardioverter-defibrillator (WCD), wearing it for an average of 22 h per day, and no ventricular arrhythmias had been recorded. Guideline-directed medical therapy comprised sacubitril/valsartan 24/26 mg twice daily, metoprolol succinate 47.5 mg twice daily, spironolactone 25 mg once daily, and empagliflozin 10 mg once daily; all medications were well tolerated. A follow-up cardiac MRI performed approximately 3 months after STEMI demonstrated an LVEF of 43% and extensive infarct scar in the LAD territory with transmurality of 75%–100% *(Figure 3).* Typical infarct-related late gadolinium enhancement was present in AHA segments 1–3, 7–9, and 13–15; transmurality was ∼75% in segments 1–3 and up to 100% in segments 7–9 and 13–15. No thrombus or residual shunt was detected. Coronary angiography confirmed durable patency of the drug-eluting stent with no evidence of new disease.

**Figure 4:**
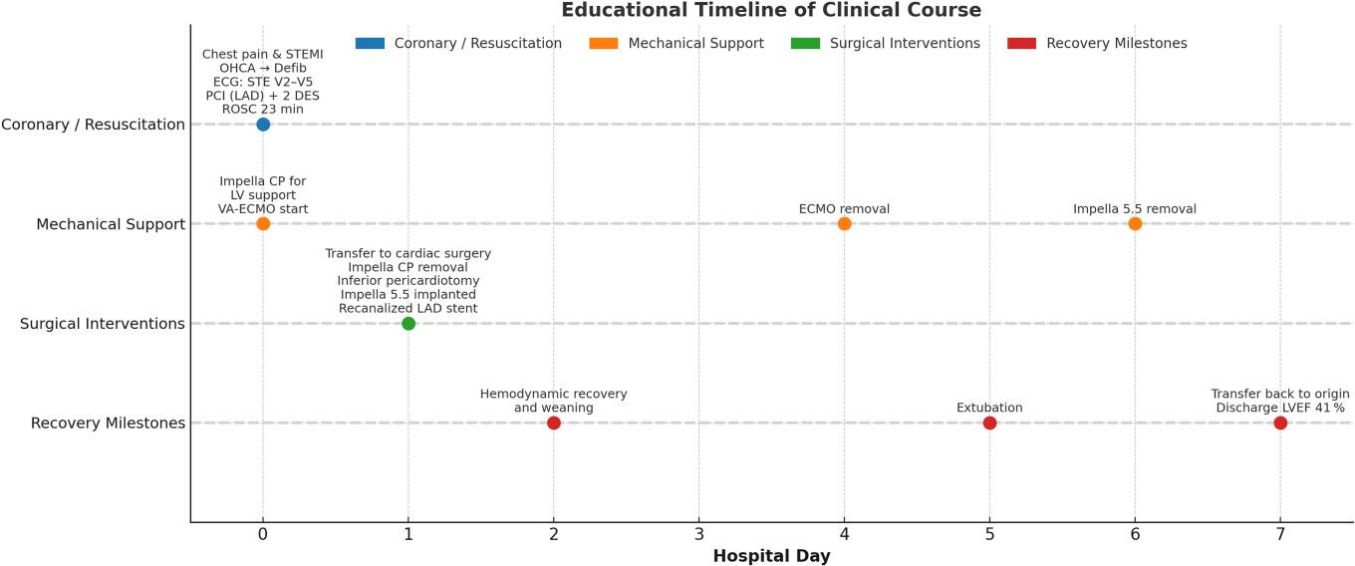
Educational timeline of clinical course.

After completing 3 months of WCD use, the patient received a subcutaneous implantable cardioverter-defibrillator (S-ICD) for primary prevention of sudden cardiac death (MADIT-ICD Benefit Score: 88, indicating high expected benefit). Follow-up TTE showed a stable LVEF of ∼43% with persistent anteroseptal and anterior akinesia. No ventricular arrhythmias were detected during the first 6 months of follow-up (*Figure 4*).

## Discussion

This case report is, to the best of our knowledge, the first to describe LV perforation secondary to Impella® CP placement in a resuscitated patient after STEMI. While the use of MCS devices, including percutaneous microaxial flow pumps, has increased in CS complicating ACS, high-quality randomized data supporting their use remain limited.

The recent DanGer-SHOCK (Danish-German Cardiogenic Shock) trial randomized 360 noncomatose patients with STEMI and CS to receive either a microaxial flow pump or standard care.^8^ The trial demonstrated a 26% relative reduction in all-cause mortality at 180 days (HR, 0.74 [95% CI, 0.55–0.99]; *P* = 0.04; absolute risk reduction, 12.7%; number needed to treat = 8) with the microaxial flow pump.^8^ This finding and the results of the ECLS-Schock trial influenced the 2025 American Heart Association (AHA) ACS guidelines, which now assign a Class IIa recommendation for microaxial intravascular flow pumps in selected patients with STEMI and severe or refractory CS to reduce mortality and a Class III recommendation for routine use of IABP or ECMO.^11^ However, the increased risk of complications—such as bleeding, limb ischaemia, and renal replacement therapy—seen in the DanGer-SHOCK trial necessitates careful patient selection and risk-benefit assessment.^11^

In STEMI, the risk of complications following Impella placement is particularly concerning. The histopathologic changes associated with transmural infarction make ventricular instrumentation more hazardous.^12^ In our case, the combination of a critically weakened, friable, necrotic myocardium from a large acute LAD infarction and the mechanical stress from repetitive resuscitative efforts, including CPR and microaxial pump placement, likely acted synergistically to cause the LV free-wall perforation. The Impella® waveform abnormality and CT evidence of a focal perforation with only minimal effusion are consistent with a device exiting through infarct-weakened tissue. We acknowledge the difficulty in isolating a single aetiology; however, the extensive transmural LAD scarring seen on the follow-up cardiac MRI, combined with the fact that the device was not repositioned prior to the event, suggests that infarct-related myocardial fragility was the critical predisposing factor. The resulting Impella malposition is most plausibly secondary to perforation in this compromised myocardium.

The fact that no macroscopic rupture tract was identified intra-operatively, despite the CT evidence of focal perforation, warrants consideration of alternative mechanisms. It is conceivable that the initial myocardial rupture, secondary to the acute STEMI, was contained by organizing tissue or a self-limiting ‘pseudomembrane’. The Impella device may have subsequently exited through this pre-existing, contained defect, explaining the minimal pericardial effusion seen on CT. While this pseudo-aneurysm mechanism remains conjectural, it is a crucial differential diagnosis in similar complex cases, emphasizing that Impella placement in the setting of acute MI is a high-risk procedure regardless of whether the rupture is acute or evolving. According to the FDA MAUDE database, 50 cases of Impella-related ventricular perforation were reported between 2009 and 2021, with repositioning of the device accounting for 24% (*n* = 12) of cases.^9^ Infarcted myocardial tissue was also identified as an underlying factor in 24% of cases.^9^

Clinicians should maintain a high index of suspicion for Impella-induced LV perforation in patients who develop a new pericardial effusion, changes in the Impella monitor’s positional waveform, or worsening heart failure.^13^ Routine transthoracic (TTE) or transoesophageal echocardiography (TEE) in the ICU is crucial for early detection of positional abnormalities and complications.^14^

This case underscores a rare but potentially fatal complication of Impella placement. High-risk patients require continuous haemodynamic monitoring to assess treatment response and detect complications promptly. Proper device handling, as per manufacturer guidelines, is essential to minimize risks. To help avoid future mechanical complications, specific implantation best-practices are provided (see Supplementary material online, *Figure S5*). Additionally, multidisciplinary collaboration between medical specialties plays a critical role in managing complications effectively. Ongoing and future randomized clinical trials will provide further evidence to guide the optimal use and timing of Impella therapy in cardiogenic shock.

### Limitations

Limitations include the lack of intraprocedural TEE at implantation and the absence of acute-phase cardiac MRI; the cMRI was obtained at ∼3 months and thus documents the extent of irreversible injury rather than the acute rupture itself.

### Patient perspective at follow-up (verbatim)

**‘**I feel lucky to still be able to see my children and my wife every day. My life has changed since what happened almost 3 months ago. I’m taking my medication carefully and coming to every follow-up as ordered.’

## Lead author biography


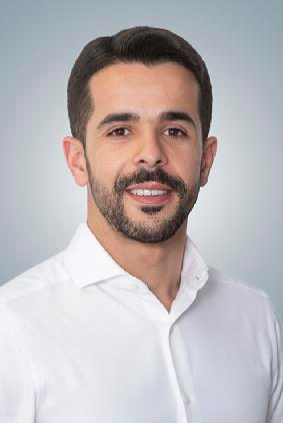


I am Omar Hajji, a cardiology resident at the University Medical Center Mannheim, Germany, with interests in cardiology, emergency medicine, and critical care. My research focuses on cardiogenic shock in acute coronary syndromes. Through case documentation and clinical research, I aim to improve the understanding and management of complex cardiovascular conditions.

## Supplementary Material

ytag050_Supplementary_Data

## Data Availability

The data underlying this article will be shared on reasonable request to the corresponding author.
