# Supplementary material for: Left ventricular perforation following impella® CP placement in a resuscitated STEMI patient with cardiogenic shock: a rare complication and case report
Source: Eur Heart J Case Rep. 2026 Jan 28;10(2):ytag050. doi: 10.1093/ehjcr/ytag050 (PMC12908187; doi:10.1093/ehjcr/ytag050)
Supplement: ytag050_Supplementary_Data [file ytag050_supplementary_data.zip › Supplementary_Legends.docx]

**Supplement Material**

**Video 1:** Coronary Angiography showing the left anterior descending (LAD) Occlusion during ongoing CPR

**Video 2:** Percutaneous Coronary Intervention (PCI) of the LAD during ongoing CPR

**Video 3:** Coronary Angiography of the Right Coronary Artery (RCA) during ongoing CPR

**Video 4:** Echocardiography at Discharge showing improved left ventricular ejection fraction (LVEF) with anteroseptal and anterior Akinesia
