# Supplementary material for: Left ventricular perforation following impella® CP placement in a resuscitated STEMI patient with cardiogenic shock: a rare complication and case report
Source: Eur Heart J Case Rep. 2026 Jan 28;10(2):ytag050. doi: 10.1093/ehjcr/ytag050 (PMC12908187; doi:10.1093/ehjcr/ytag050)
Supplement: ytag050_Supplementary_Data [file ytag050_supplementary_data.zip › Figure 5 - Impella_Implantation-Tips-.pdf]

1 Positioning under fluoroscopy
– Use pigtail catheter to mark LV apex

2 Confirm tip just above mitral valve
– LAO-cranial cine & contralateral marker

3 Verify console parameters
– P-level ≤8, purge 500-800 mmHg, flow ≥3.5 L min^{-1}

4 Checklist before leaving lab
– No alarms, stable waveform, ACT ≥250 s

Figure 5: Impella Implantation Tips
